# Alterations of Nigral Dopamine Levels in Parkinson’s Disease after Environmental Enrichment and PACAP Treatment in Aging Rats

**DOI:** 10.3390/life11010035

**Published:** 2021-01-08

**Authors:** Adel Jungling, Dora Reglodi, Gabor Maasz, Zita Zrinyi, Janos Schmidt, Adam Rivnyak, Gabor Horvath, Zsolt Pirger, Andrea Tamas

**Affiliations:** 1 MTA-PTE PACAP Research Team, Department of Anatomy, Medical School, University of Pecs, 7624 Pecs, Hungary; adel.jungling@aok.pte.hu (A.J.); dora.reglodi@aok.pte.hu (D.R.); adam.rivnyak@aok.pte.hu (A.R.); gabor.horvath@aok.pte.hu (G.H.); 2 MTA-OK BLI NAP_B Adaptive Neuroethology, Department of Experimental Zoology, Balaton Limnological Institute, MTA-CER, 8237 Tihany, Hungary; maasz.gabor@okologia.mta.hu (G.M.); zrinyi.zita@okologia.mta.hu (Z.Z.); pirger.zsolt@okologia.mta.hu (Z.P.); 3 Institute of Biochemistry and Medical Chemistry, Medical School, University of Pecs, 7624 Pecs, Hungary; janos.schmidt@aok.pte.hu

**Keywords:** Parkinson’s disease, 6-OHDA, aging, enriched environment, PACAP

## Abstract

The neuroprotective effects of environmental enrichment and PACAP (pituitary adenylate cyclase-activating polypeptide) are well-described in Parkinson’s disease. The aim of our study is to investigate the beneficial effects of these factors in aging parkinsonian rats. Newborn Wistar rats were divided into standard and enriched groups according to their environmental conditions. Standard animals were raised under regular conditions. During the first five postnatal weeks, enriched pups were placed in larger cages with different objects. Aging animals received (1) saline, (2) 6-hydroxidopamine (6-OHDA), or (3) 6-OHDA + PACAP injections into the left substantia nigra (s.n.). On the seventh postoperative day, the left and right s.n. were collected. The s.n. of young and aging unoperated animals were also examined in our experiment. We determined the dopamine (DA) levels by the HPLC-MS technique, while the sandwich ELISA method was used to measure the Parkinson disease protein 7 (PARK7) protein levels. In healthy animals, we found an age-related decrease of DA levels. In aging parkinsonian-enriched rats, the operation did not result in a significant DA loss. PACAP treatment could prevent the DA loss in both the standard and enriched groups. All injured PACAP-treated rats showed remarkably higher protective PARK7 levels. The protective effect of PACAP correlated with the increase of the DA and PARK7 levels.

## 1. Introduction

The second most common neurodegenerative disorder, Parkinson’s disease, affects around one percent of the elderly population over the age of sixty [1,2]. Parkinson’s disease is characterized by progressive motor and mild neuropsychiatric symptoms due to the loss of dopaminergic neurons of the substantia nigra (s.n.) pars compacta [3,4]. The decreased dopamine (DA) level in the nigrostriatal system leads to the most characteristic motor signs: tremor, rigidity, and bradykinesia [5,6]. Although the definite pathophysiological cause of the disease is still unknown, it is proposed that oxidative stress, neuroinflammation, protein misfolding, and mitochondrial disfunction may play a role in the neurodegeneration [7,8]. The mutation of genes responsible for mitochondrial homeostasis can lead to familial forms of Parkinson’s disease: α-synuclein, parkin, phosphatase and tensin homolog-induced kinase 1 (PINK1), and Parkinson disease protein 7 (PARK7) [9,10,11,12]. PARK7 (also known as DJ-1) is a chaperone protein belonging to the peptidase C56 family. Several studies have described that it prevents oxidative stress-induced apoptosis, leading to a neuroprotective effect [13,14]. Additionally, environmental factors such as age, gender, physical trauma, and toxic effects (tobacco and pesticides) are also associated with the development of Parkinson’s disease [15,16]. Age is an extremely important factor in the development of the disease [17]. Both its incidence and prevalence increase steadily with age. Its mortality also raises 10 years after the diagnosis compared to healthy individuals [18].

Although several pharmacological and nonpharmacological therapies are available to ameliorate the symptoms of Parkinson’s disease, so far, there is no solution that would counteract the cause of the disease [19]. In the lack of an ideal therapy, several ongoing researches are focused on new neuroprotective agents with the potential of saving the dopaminergic cells of s.n., such as polyphenols, antibiotics, and neuropeptides [3]. One focus of our current investigation is the neuropeptide PACAP (pituitary adenylate cyclase-activating polypeptide). It has numerous beneficial effects in models of neurodegeneration: it serves as an antiapoptotic, anti-inflammatory, and an antioxidant agent [20]. The presence of PACAP receptor mRNA and, also, PACAP expression is described in the substantia nigra [21,22], while PAC1 receptors are in the striatum, the end of the nigrostriatal pathway [23]. These suggest its potential protective impact on dopaminergic neurons [20]. An age-related decrease is described in the cerebral expression of the peptide, which might lead to an increased vulnerability of the aging population to neurodegenerative diseases [24,25,26]. One of the most widespread models of Parkinson’s disease in rodents is created by a unilateral lesion of the s.n. with 6-hydroxidopamine (6-OHDA). Our research group has described the protective effects of exogenous PACAP following a 6-OHDA-induced lesion. PACAP therapy led to less severe hypokinetic symptoms, the improvement of asymmetrical signs, and reduced dopaminergic cell loss in young rats [27,28]. In aging animals, similar results were found concerning the number of dopaminergic cells. Although PACAP treatment enhanced motor recovery, its effect was less strong compared to young animals [29]. Recently we demonstrated that the peptide also exerts neuroprotective functions by resulting in less decreased nigral DA levels following a 6-OHDA-induced lesion [30]. 

In addition topharmacological therapy, it has been described that environmental factors can alter both the prevalence and outcome of Parkinson’s disease [31]. In laboratories, enriched circumstances can model positive environmental factors and a stimuli-rich environment. The beneficial effects of an enriched environment are reinforced by thousands of studies since the first description of Donald Hebb [32]. Our research group has shown that environmental enrichment has protective effects in neonatal and adult retina lesions [33,34], in neonatal asphyxia, and in glutamate-induced toxicity [35,36]. In the case of Parkinson’s disease, our research group provided evidence for the neuroprotective effect of a postnatal-enriched environment in adult rats [37]. In the last few decades, numerous studies have proven the beneficial impact of environmental enrichment in different models of Parkinson’s disease [38]. Almost all these experiments were performed on young/adult animals aged between eight weeks to seven months. Inducing Parkinson’s disease in aging animals would give us a good model of the human disease. The oldest rats in studies examining the beneficial effects of enrichment in Parkinson’s disease were adults [39,40,41,42].

Based on these investigations, the aim of our current experiment was to set up a human-relevant model of Parkinson’s disease by inducing dopaminergic cell loss in aging rats by a unilateral nigral lesion. We examined the effects of two possible protective factors: the neuropeptide PACAP and a nonpharmacological treatment strategy environmental enrichment. The DA and PARK7 protein levels were evaluated in healthy, unoperated, saline- (0.9% NaCl), 6-OHDA-, and 6-OHDA + PACAP-treated animals both in standard and enriched groups to explore whether an early enriched environment still has a potential protecting effect later in life and whether there is an additional effect with the well-known protective agent, PACAP.

## 2. Materials and Methods

### 2.1. Experimental Animals and Environmental Enrichment

Wistar rats bred at the Department of Anatomy (Medical School, University of Pecs, 7624 Pecs, Hungary) were used in our studies (*n* = 75). Animals were kept under a 12-h light–dark cycle and provided with food and water ad libitum. All experimental animals were kept in the same room, under the same illumination and other outside environmental conditions, to avoid any environmental effects other than our standard/enriched cages. Animal housing, care, and all experimental procedures were performed in accordance with institutional guidelines under approved protocols (No: BAI/35/55-2/2017, University of Pecs following the European Community Council directive). Efforts were made to reduce the suffering and the number of animals in the experiments.

In concordance with our earlier studies, animals were divided into standard and enriched groups based on their environmental conditions [33,35,37]. Standard animals (*n* = 43) were kept in standard cages (43 cm× 30 cm× 20 cm) under regular conditions. Pups of the enriched group (*n* = 32) were placed in larger cages (88 cm × 50 cm × 44 cm) during the first five postnatal weeks. Our enriched cages were supplemented with toys, objects, running tunnels, and rotating rods of different shapes, sizes, and materials to provide multiple sensorimotor stimuli. Half of the toys were changed daily for cognitive stimuli. After this five-week-long period, enriched animals were also kept under regular circumstances.

### 2.2. Healthy, Unoperated Animals

The DA and PARK7 protein levels of the s.n. of healthy, unoperated (*n* = 19) were also measured in our experiment. We collected samples from both young (3 to 4 months) and aging (14 to 18 months) rats in order to compare the baseline level of these substances of young and aging and standard and enriched animals.

### 2.3. Saline, 6-OHDA, and PACAP Treatments

Parkinson’s disease was induced similarly to our previous studies [28,31,37] in aging 14–18-month-old animals. Based on the treatment, both standard and enriched animals were divided randomly into three groups. One group of animals received 2-µL physiological saline (0.9% NaCl) into the left s.n. (from Bregma point: 5.5-mm posteriorly, 2.5-mm left, and 8-mm ventrally). Another group of animals was treated with unilateral injections of 2-µL 6-OHDA (5 µg/µL, Sigma, Budapest, Hungary) to the same location to induce Parkinson’s disease. The third group received an additional local PACAP treatment (2 µL, 1-µg/µL concentration) [43,44] directly after the 6-OHDA injections (6-OHDA + PACAP). Injections were delivered with a Hamilton syringe, each time over a 2-min period, and the needle was left in place for another 2 min. The right side of the animals was always left undamaged, serving as the control side.

### 2.4. Measurement of Dopamine and PARK7 Levels

On the 7th postoperative day, brains were collected and frozen to −80 °C. Then, samples of both the right and left s.n. were taken with the help of Brain Matrix (Braintree Scientific Inc., Braintree, MA, USA) at −15 °C. Dopamine levels of the s.n. were measured by the HPLC-MS (high-performance liquid chromatography-mass spectrometry) technique. Parallel with these measurements, the level of the PARK7 protein was also determined with the sandwich ELISA (enzyme-linked immunosorbent assay) method. These evaluations were performed similarly to our earlier study, following the exact descriptions of the manufacturers [30].

All data are expressed as mean ± S.E.M. Results were analyzed with GraphPad Prism 8.0 (GraphPad Software, San Diego, CA, USA), using an ordinary one-way ANOVA test, followed by Tukey’s multiple comparisons test or a two-sample *t*-test. Differences were considered significant at *p* < 0.05.

## 3. Results

### 3.1. Dopamine Levels of the Substantia Nigra

**Figure 1:**
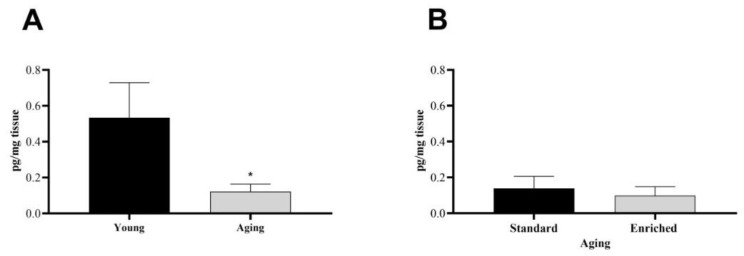
Dopamine content of the substantia nigra (s.n.) of (**A**) young and aging and (**B**) aging standard and enriched animals. Values are given in pg/mg tissue, as mean ± S.E.M. A two-sample *t*-test was used for the statistical analysis. * *p* < 0.05 versus young animals.

First, we examined the baseline dopamine levels of the s.n. in healthy, unoperated animals (*n* = 11). In this case, we added the DA levels of the left and right s.n., since, in the control group, we did not expect significant differences between the two sides of the brain (Figure 1). We found a significantly lower level of DA in the s.n. of aging rats compared to young, healthy individuals (Figure 1A). In the aging group, there was no difference in the nigral DA content of the standard and enriched rats (Figure 1B).

**Figure 2:**
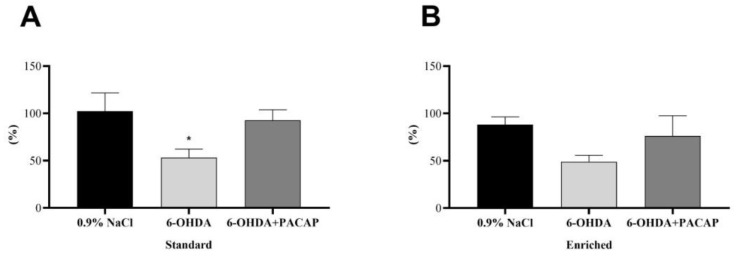
Percentage of the dopamine contents of the s.n. of the injured side compared to the undamaged, control side of (**A**) standard and (**B**) enriched rats. Values are given in percentage, as mean ± S.E.M. One-way ANOVA followed by Tukey’s multiple comparisons test was used for the statistical analysis. * *p* < 0.05 versus 0.9% NaCl-treated animals of the same group. 6-OHDA: 6-hydroxidopamine and PACAP: pituitary adenylate cyclase-activating polypeptide.

In the case of parkinsonian aging animals (*n* = 48), the percentage of DA concentration on the lesioned (left) side was compared to the control, undamaged s.n. of each animal (Figure 2). In the standard group (*n* = 28), our studies revealed a significant decrease of DA levels following 6-OHDA injections compared to saline-treated animals of the same group, confirming the success of the unilateral nigral lesion. When rats were treated with PACAP after receiving 6-OHDA, this drop could not be observed (Figure 2A); the DA content of the s.n. was only reduced to 92.73% ± 11.14%. In the enriched group (*n* = 20), early environmental stimuli could prevent the significant DA loss in our Parkinson model. The DA level of the PACAP-treated group (6-OHDA + PACAP) was 27.14% higher than that of the 6-OHDA-treated animals (Figure 2B). In both the standard and enriched animals, PACAP treatment resulted in only a 10–12% decline of DA levels compared to saline-treated animals of the same group.

### 3.2. PARK7 Levels of the Substantia Nigra

**Figure 3:**
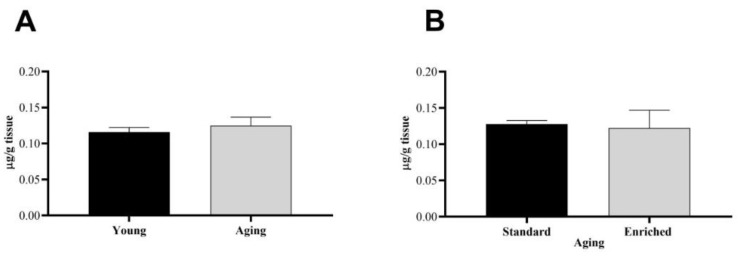
Parkinson disease protein 7 (PARK7) protein content of the s.n. of (**A**) young and aging and (**B**) aging standard and enriched animals. Values are given in µg/g tissue, as mean ± S.E.M. A two-sample *t*-test was used for the statistical analysis.

The PARK7 levels of the s.n. were also quantified in our non-parkinsonian healthy group (*n* = 8) (Figure 3). The baseline levels of the protective protein did not show an age-dependent change; there were no significant differences between young and aging animals (Figure 3A). Furthermore, early environmental enrichment did not influence the level of PARK7 protein either (Figure 3B).

**Figure 4:**
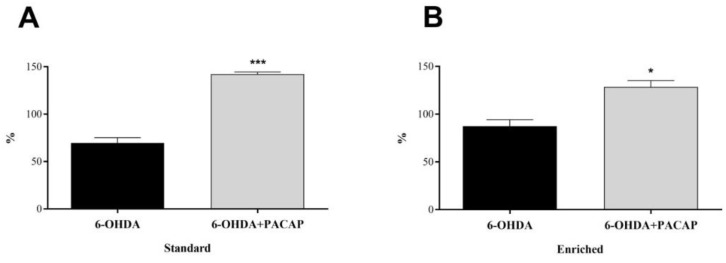
Percentage of PARK7 content of the s.n. of the injured side compared to undamaged, control side of (**A**) standard and (**B**) enriched rats. Values are given in percentage, as mean ± S.E.M. A two-sample *t*-test was used for the statistical analysis. (**A**) *** *p* < 0.001 versus 6-OHDA-treated animals of the same group. (**B**) * *p* < 0.05 versus 6-OHDA-treated animals of the same group.

In parkinsonian aging animals (*n* = 8), we calculated the percentage of PARK7 levels of the left, injured s.n. compared to the right, undamaged side (Figure 4). Although there were no significant differences between the standard and enriched rats, a 6-OHDA injection led to a decrease of PARK7 protein content to 69.47% of the undamaged, control side in standard animals, while only to 87.31% in enriched animals. In both animal groups, PACAP caused a significant elevation of PARK7 proteins compared to 6-OHDA-treated animals of the same group.

## 4. Discussion

Considering our aging population, the prevalence of Parkinson’s disease is expected to be doubled in the next two decades [45]. More than two hundred years after the first description of the disease, despite all efforts to find a cure and accumulation of experimental data, an effective therapy of Parkinson’s disease is yet to be developed. In our current experiment, we examined whether early environmental enrichment and the neuropeptide PACAP could intervene in the pathophysiology of the disease.

In the first part of our study, we determined the DA levels of the s.n. Our measurements in healthy, unoperated animals showed an age-related decrease of DA levels, which is not surprising, since aging affects the neurochemistry of the dopaminergic system. It is well known that neurons of the s.n. pars compacta are gradually degenerated with age, leading to reduced dopamine metabolism [46,47]. Earlier, our research group compared the behavioral and morphological consequences of a 6-OHDA-induced lesion in young and aging rats. Before the operation, aging animals covered less distance and showed less rearing activity than young individuals, which could be correlated with the age-related dopaminergic decline. In aging animals, the lesion resulted in a significant reduction of motor activity and a slightly more dopaminergic cell loss compared to young animals [29]. Since aging is the major risk factor of Parkinson’s disease, aging animals would be the most appropriate models to study experimental therapies for Parkinson’s disease. Thus, it is of importance that our current research focuses on the changes of dopaminergic systems of aging rats.

When inducing Parkinson’s disease with a unilateral injection of 6-OHDA, we investigated the possible neuroprotective potential of two factors that are proven to be effective in young parkinsonian animals: environmental enrichment and PACAP. Numerous studies have described that an enriched environment is able to alter the pathology and symptoms of Parkinson’s disease [38]. Enrichment results in beneficial cellular and biochemical effects, such as a decreased loss of tyrosine-hydroxylase (TH)-positive dopaminergic cells, increased GFAP (glial fibrillary acidic protein)-positive cells, reduced DAT (dopamine transporter) and VMAT2 (vesicular monoamine transporter 2) expression, and increased levels of neuroprotective agents, like GDNF (glial cell-derived neurotrophic factor) and BDNF (brain-derived neurotrophic factor). A potential protective mechanism can also be the elevation of PACAP neuropeptide levels. It has been described that a three-week-long enrichment results in the increase of PACAP27- and PACAP38-like immunoreactivities in different areas of the adult rat brain [48].

The majority of the published experiments regarding PD exposed animals to an enriched environment before, concurrently, or directly after inducing dopaminergic cell loss [38], although, in our previous article, we suggested that early, postnatal environmental enrichment might also have an effect later in life [37]. In the early phases of life, environmental factors have important roles in the development of the nervous system. The nervous system has exceptional plasticity at this age; thus, both harmful and positive effects might have long-lasting significance [35,36,49,50]. Our research group’s previous experiment provided evidence for the neuroprotective effects of the postnatal enriched environment of Parkinson’s disease in adult rats [37]. In three-month-old animals, we evaluated the motor symptoms and tyrosine-hydroxylase (TH)-positive cell loss in the s.n. We showed that enriched animals produced less severe hypokinetic symptoms compared to their standard mates. Due to enrichment, a recovery was visible in the number of free rearings 10 days after the operation, and the distance covered by the animals did not decrease significantly after 6-OHDA treatment. In young adult rats raised in a standard environment, we found a significant loss of dopaminergic neurons compared to saline-treated animals; in contrast, this loss could not be observed in enriched animals; thus, early enrichment could rescue the dopaminergic cells of the s.n. after a 6-OHDA-induced lesion. These results correlate well with our current findings that the toxin only caused a significant decrease of the nigral DA level in the case of standard animals. Animals raised under enriched conditions were more protected against the toxin; the operation did not result in a significant DA loss compared to saline-treated animals of the same group. Although there was no significant difference between 6-OHDA-treated standard and enriched animals, DA levels dropped by 48.94% in the standard group but only by 39.23% in the enriched group. This slight difference in the DA levels suggests a better ability of compensation and might lead to better motor performance in enriched animals. Similar results were found in adult animals, where a minor difference in the dopaminergic cell number resulted in improved motor recovery [37]. In the case of healthy, unoperated aging rats, postnatal enrichment did not have an effect on the DA levels, but our results indicate that it is able to exert a mild neuroprotective effect in a neurodegenerative condition.

In aging parkinsonian groups, the PACAP treatment could counteract the toxin-induced lesion, since it prevented the DA loss. This effect was more prominent in the standard group, because the 6-OHDA lesion originally led to a significant DA loss in that group. These results provide new data about the neuroprotective effect of PACAP in aging animals after we demonstrated recently in young animals that an intranigral PACAP co-treatment could attenuate the decrease of DA levels in the s.n. following 6-OHDA injection [30]. These results are in concordance with numerous studies reporting the potential therapeutical effects of PACAP inhibiting pathological processes and improving the symptoms of Parkinson’s disease [24,51,52]. The toxin 6-OHDA causes a mitochondrial dysfunction of DA-producing neurons of the s.n. [53]. It increases the level of reactive oxygen species (ROS), leading to elevated levels of proapoptotic agents, such as cytochrome c and caspases, which, finally, result in neuronal damage [3,54,55]. In most cases, the neuroprotective effects of PACAP are exerted on the G-protein-linked PAC1 receptor. When PACAP binds to the PAC1 receptor, it activates the adenylate cyclase enzyme, increasing the level of cAMP (cyclic adenosine monophosphate). cAMP consequently activates protein kinase A, in which the cell signaling cascade is the main pathway responsible for PACAP-induced neuroprotection [56,57]. PACAP signaling leads to the inhibition of proapoptotic and elevation of antiapoptotic factors [52,58]. PACAP is reported to influence several growth factors and cytokines to achieve its beneficial effects. It stimulates the expression of BDNF (brain-derived neurotrophic factor) in rat cerebral cortex cells in vitro [59] and other trophic factors, like CTNF (ciliary neurotrophic factor) and LIF (leukemia inhibitory factor). The neuropeptide is also able to inhibit the release of proinflammatory cytokines like TNF and interleukin 1 (IL-1) [60]. PACAP is also capable of suppressing the production of microglia-derived reactive oxygen species [61]. Additionally, it activates anti-inflammatory pathways and antioxidant molecules, which strengthen its potency to counteract neuronal damage [62]. Furthermore, the neuropeptide is shown to play a role in cellular and tissue aging [63]. Relevant to our current study is the influence of PACAP on catecholamine, especially on DA synthesis. The process of dopamine synthesis starts by the enzyme tyrosine-hydroxylase (TH) forming L-DOPA (levodopa, or L-3,4-dihydroxyphenylalanine) from tyrosine; then, L-DOPA carboxylase (DDC) converts L-DOPA to dopamine. PACAP is able to activate TH in several parts of the central nervous system (CNS). It elevates the quantity of the TH enzyme and the number of TH-positive neurons in the mesencephalon [21,64], and it also increases the exocytosis of DA [65]. As a consequence, PACAP has the capacity to potentially increase the DA levels.

In order to find out the protective mechanisms of PACAP in our model, we examined the proteomic changes of the s.n. potentially leading to higher DA levels. In our current study, we focused on the exact quantitative determination of the PARK7 (DJ-1) chaperon protein based on the findings of the study of Maasz and coworkers in the same animal model [30], who evaluated the quality and quantity of the total protein content of the s.n. following a 6-OHDA-induced lesion and PACAP treatment. The quantitative analysis by the nano-LC-MS method focused on 95 different Parkinson’s disease-relevant proteins (for example, the myelin basic protein, thiosulfate sulfur transferase, and PARK7); among those, only one, PARK7, presented significantly different levels due to treatment. PARK7 is shown to be one of the genes, the mutation of which leads to a familial form of Parkinson’s disease with autosomal recessive inheritance [66]. In this form, the symptoms appear earlier in life, although with slower progression. Observations of DJ-1 knockout mice declare the essential role of this protein in the dopaminergic system [67], since these mice show reduced striatal DA overflow and decreased motor activity in the open field test but without change in the number of DAergic cells in the s.n. or striatal DA levels [68,69]. However, there are lines of DJ-1-deficient mice, which show TH-positive neuron loss in the ventral tegmental area [70] or unilateral loss of DA-producing neurons of the s.n. leading to bilateral degeneration of the nigrostriatal system in aging animals [71]. Furthermore, Kyser and coworkers described the presence of several motor and non-motor-behavioral alterations in a PARK7 knockout genetic rat model [72]. In addition, PARK7 is accepted to have a role in antioxidative processes, since the downregulation of the protein leads to oxidative stress-induced cell death, while its overexpression could rescue a neuronal cell line against hydrogen peroxide and 6-OHDA toxicity [73,74]. In addition, the knockdown of PARK7 in neuroblastoma cells resulted in a susceptibility to hydrogen peroxide-, MPP^+^-, and 6-OHDA-induced cell death [75]. Recently, Guzman and coworkers also proved the cytoprotective role of PARK7 in dopaminergic neurons. The deletion of PARK7 increased the basal mitochondrial oxidative stress in s.n. neurons by compromising mitochondrial uncoupling processes [76]. If the loss of function of PARK7 affects the pathogenesis of PD, it may be suggested that its activation offers beneficial effects. Several specific mechanisms describe how PARK7 is able to protect dopaminergic cells. It is a transcriptional regulator protecting mitochondria from oxidative stress, a chaperone, and it also works as a protease [77]. It is able to alter several transcriptional factors in oxidative stress. It prevents Nrf2 (nuclear factor erythroid 2-related factor) from associating with its inhibitor, thus allowing it to properly regulate antioxidant transcriptional processes [13]. The PI3K (phosphoinositide 3-kinase)/PKB (protein kinase B) signaling cascade is an important pathway leading to cell growth and preventing cell death in the case of numerous neurodegenerative disorders [78]. This pathway can be inhibited by the lipid phosphatase PTEN (phosphatase and tensin homolog). In the case of oxidative stress conditions, PARK7 is able to form a complex with PTEN, thus restricting its inhibition on the protective PI3K/PKB pathway [79,80]. The p53 protein plays a role in the induction of apoptosis. It has been shown that PARK7 suppresses the transcription of p53, consequently preventing apoptosis [81,82]. PARK7 is mostly found in the cytoplasm of DAergic neurons, but ROS-induced oxidative stress leads to the overproduction and relocation of PARK7 to the mitochondria, suggesting that it can also exert its protective effect in that organelle [83]. Bcl-xL (B-cell lymphoma extra-large) is a mitochondric transmembrane molecule belonging to the Bcl-2 (B-cell lymphoma 2) family. It is an antiapoptotic agent inhibiting the release of cytochrome c from the mitochondria [84]. Under oxidative stress, PARK7 binds to Bcl-xL and stabilizes it to prevent its degradation [85]. Other possible mechanism of its protection is that it inhibits the aggregation of α-synuclein, decreasing Lewy-body formation, which is a typical pathology of both hereditary and sporadic Parkinson’s disease [14]. The expression of PARK7 can decrease the dimerization of α-synuclein [86], and its overexpression leads to reduced α-synuclein levels in vitro. It can also inhibit the accumulation of α-synuclein by regulating chaperone-mediated autophagy [87]. It is proven that the PARK7 protein upregulates TH gene expression in humans [88] and, also, directly activates TH and DDC, leading to DA synthesis [89].

Similar to our current model, Inden and coworkers induced Parkinson’s disease with a unilateral 6-OHDA injection into the s.n. A co-injection with a recombinant PARK7 protein protected against the loss of TH-immunoreactive cells and the drug-induced rotational asymmetry [90]. Our previous study on young parkinsonian animals revealed that the level of the PARK7 protein was significantly lower in 6-OHDA-treated rats compared to healthy control animals, while a PACAP co-treatment almost completely restored the PARK7 level [30]. In our present research, we reported that neither age nor environmental circumstances influenced the level of PARK7 protein in healthy, unoperated animals. In parkinsonian rats, PACAP has the same effect in aging and young adults: it causes a significant increase of the protective PARK7 protein in the injured s.n. Both the standard and enriched PACAP-treated animals showed remarkably raised PARK7 levels; the protein level was even above 100%, which suggests that this elevation could be a compensatory defense mechanism activated by PACAP therapy.

**Figure 5:**
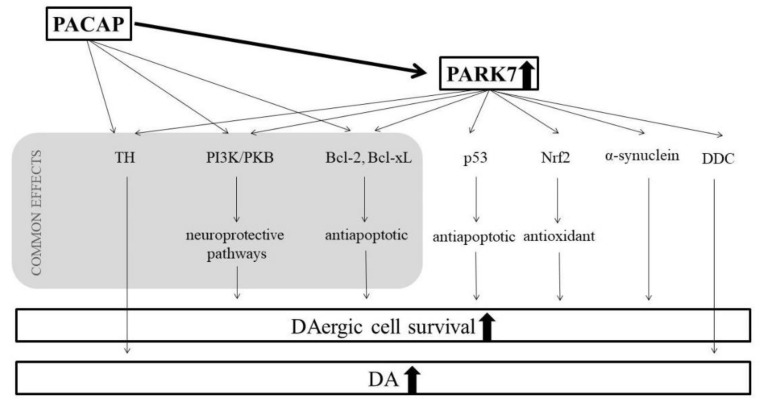
Possible connections between the protective mechanisms of PACAP and PARK7 proteins.

Our results suggest a clear connection between the effects of PACAP and the PARK7 protein (Figure 5). Their protective mechanisms meet at several points; they exert effects on various common pathways. It is well known that PACAP—similarly to the PARK7 protein—also activates the PI3K/PKB pathway, leading to increased neuronal survival [91,92]. Another common point is their antiapoptotic effects through Bcl-xL. In the case of glutamate-induced apoptosis, PACAP increases the expression of Bcl-2 and Bcl-xL by stimulating the PKB/14.3.3 protein/Bad (Bcl-2 associated death promoter) cascade [93]. The Bcl-2-involved neuroprotection of PACAP was also described in focal ischemia in mice and alcohol toxicity in rats [94,95]. Finally, our novel observation in aging parkinsonian rats is that the protective effect of PACAP correlates well with the increase of the DA and PARK7 protein levels. So far, no studies have explored PACAP’s potential additional effects with an enriched environment in Parkinson’s disease. It has been described that environmental enrichment itself is capable of increasing the brain PACAP levels [48], and we explored mildly higher DA and PARK7 levels after 6-OHDA treatment in enriched animals. However, we observed that postnatal environmental enrichment could not strengthen the effect of PACAP therapy, since we did not find significant differences between PACAP-treated standard and enriched rats.

## 5. Conclusions

The epidemiology of Parkinson’s disease draws further attention to the importance of carrying out experiments in aging animals. Our aging model is the first to provide evidence for the potential protective effects of early-life enriched housing conditions in the case of Parkinson’s disease. We showed that the early enriched environment can still have an effect on a neurodegeneration-induced dopamine loss later in aging individuals. This draws further attention to noninvasive, nonpharmaceutical methods in the prevention of Parkinson’s disease and emphasizes the importance of a stimuli-rich environment in childhood. Our findings regarding the efficiency of PACAP reinforce studies describing its potential therapeutic effects in PD.

## Data Availability

The data presented in this study are available in the article, there is no supplementary data.
